# AT(N), neuronal and glial biomarkers in young‐onset neurodegenerative disorders and psychiatric disorders and their associations with neuropsychiatric symptoms and cognition

**DOI:** 10.1002/alz.093536

**Published:** 2025-01-09

**Authors:** Wei‐Hsuan Chiu, Anita M Y Goh, Dhamidhu Eratne, Dennis Velakoulis, Samantha M Loi

**Affiliations:** ^1^ The University of Melbourne, Parkville, VIC Australia; ^2^ The University of Melbourne, Melbourne, VIC Australia; ^3^ Melbourne Neuropsychiatry Centre, University of Melbourne and Melbourne Health, Parkville, VIC, VIC Australia; ^4^ Edith Cowan University, Perth, Western Australia Australia; ^5^ Neuropsychiatry Royal Melbourne Hospital, Parkville, VIC Australia; ^6^ National Ageing Research Institute, Melbourne, VIC Australia; ^7^ National Dementia Diagnostics Laboratory, The Florey Institute of Neuroscience and Mental Health, Parkville, VIC Australia; ^8^ Neuropsychiatry, The Royal Melbourne Hospital, Parkville, VIC Australia; ^9^ University of Melbourne, Parkville, VIC Australia; ^10^ Melbourne Neuropsychiatry Centre, University of Melbourne and Melbourne Health, Parkville, VIC Australia

## Abstract

**Background:**

It is clinically a challenging task to accurately differentiate complex young‐onset neurodegenerative disorders from psychiatric disorders which often present with similar neuropsychiatric symptoms (NPS) in their early stages. The aim of this study is to provide a more nuanced understanding of the interplay between NPS, cognition and multiple pathologies that may drive these disorders.

**Method:**

This study was conducted at the Neuropsychiatry Centre, the Royal Melbourne Hospital, Melbourne, Australia, as part of a large, multi‐site research study, the Markers in Neuropsychiatric Disorders Study. Sixty participants, including people with Alzheimer’s disease, Huntington’s disease, frontotemporal dementia, major depressive disorder and generalised anxiety disorder and together with healthy controls, were recruited and assessed for plasma biomarkers, NPS and cognition. Their plasma levels of amyloid‐beta, total‐tau (t‐tau), phosphorylated‐tau (p‐tau), neurofilament light chain protein (NfL) and glial fibrillary acidic protein (GFAP) were measured. The severity of multiple common NPS such as depression, anxiety and apathy were also measured. Cognitive functions were measured by a combination of cognitive screening tool and standardised neuropsychological assessments. General linear regressions were used to investigate the associations between biomarkers, NPS and cognition in these disorders and the differences between the two clinical groups.

**Result:**

The analysis of the collected data is ongoing. However, results from a pilot study suggest that memory recall was related to an interplay between AT(N) pathologies, and executive function was related to a neuronal pathology. The severity of NPS may moderate these relationships.

**Conclusion:**

Young‐onset neuropsychiatric disorders, presenting with overlapping symptoms, pose a diagnostic dilemma. The results of this study provide a more nuanced understanding of the intricate interplay between NPS, cognition and biomarkers that may shed light into the differentiation between young‐onset neurodegenerative disorders and psychiatric disorders.

